# Unusual Presentation of a Posterior Reversible Encephalopathy Syndrome With Brainstem Involvement and Subarachnoid Haemorrhage

**DOI:** 10.7759/cureus.16295

**Published:** 2021-07-10

**Authors:** Antonio Navarro-Ballester, Rafael Revert- Espí

**Affiliations:** 1 Radiology Department, Hospital General Universitario de Castellón, Castellón de la Plana, ESP

**Keywords:** posterior reversible encephalopathy syndrome (pres), subarachnoid haemorrhage, brainstem lesion, high blood pressure, eye ptosis

## Abstract

Posterior reversible encephalopathy syndrome is an increasingly recognized disorder characterized by a headache, visual disturbances, and seizures. It is a reversible neurotoxic state, with multiple risk factors in which endothelial injury and compromised brain perfusion are the common characteristics. Diagnosis is usually made by cerebral magnetic resonance imaging that typically shows early-stage bilateral symmetrical parieto-occipital hyperintensities on T2 and fluid-attenuated inversion recovery (FLAIR) sequences. However, other locations have been described where the disease may appear less frequently. We describe the case of a 62-year-old man, with a medical history of hypertension, who presented with anisocoria with mydriatic non-reactive pupil and ptosis of the left eye. CT head showed a slightly hypodense brainstem, in relation to vasogenic edema. This was confirmed with magnetic resonance imaging. The angiography did not identify cerebral artery aneurysms. The symptoms and radiological findings were almost completely reversible after improving the patient's blood pressure. This case highlights a rare single presentation of posterior reversible encephalopathy syndrome associated with subarachnoid hemorrhage. A high index of suspicion, careful examination, and exploration with imaging techniques were essential to reach this diagnosis.

## Introduction

The posterior reversible encephalopathy syndrome (PRES) is a reversible clinical and radiological syndrome. Its real incidence has only been studied in the pediatric population (PRES-related hospitalizations in children of 0.04%) [1] and in patients with certain associated risk factors, such as advanced kidney disease (incidence of 0.84%) [2], or transplant patients, potentially associated with cyclosporine and tacrolimus (range, 0.34% [Kidney/Kidney-Pancreas] to 0.84% [Small Bowel]) [3]. Patients may usually present with a headache, seizures, encephalopathy, visual disturbance, or acute confusion [4]. The areas of the brain usually affected are the white matter of the cortical and subcortical parietooccipital region and frontal posterior region. Less commonly it may affect the cerebellum, brain stem, basal ganglia, and anterior cerebral hemisphere.

The etiology of PRES, in descending order of clinical frequency, can be divided into hypertension (61%), cytotoxic medications (19%), preeclampsia or eclampsia (6%), autoimmune and systemic conditions, including sepsis (< 5%) [5]. The distinctive radiological findings of PRES help for its early diagnosis and management [6].

## Case presentation

A 62-year-old man with a known past medical history of hypertension and dyslipidemia was brought by ambulance to the emergency room of our hospital. He reported a sudden onset of nausea and non-projective vomiting with partially digested food content in the last six-hour period. The patient was previously healthy and had not ingested toxins or medication. He also reported a mild holocranial headache.

During the general examination, he had a blood pressure of 190/95 mmHg with a heart rate of 47 beats per minute. Cardiac and pulmonary auscultation was normal. The abdomen was soft, depressible, and painless to palpation with no signs of peritonism. He was disoriented in time and space, showing bradypsychia and bradylalia. In addition, he began to experience a severe headache and neck pain. The neurological examination revealed a left eye down and out, pupil dilated and unresponsive to any stimuli, and ptosis, which suggested third nerve palsy.

**Figure 1:**
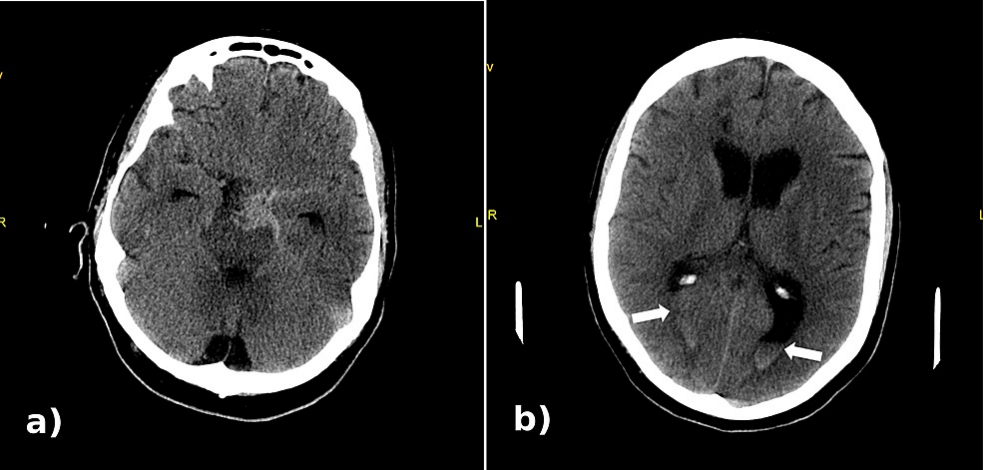
Computed tomography of the head without intravenous contrast (axial plane) a) Computed tomography of the brain shows subarachnoid hemorrhage in ambiens, crural, interpeduncular, and Sylvian cisterns. Note the hypodensity of the midbrain associated with edema. b) Hydrocephalus in lateral ventricles, blood content in the occipital horns of lateral ventricles, and mild hydrocephalous (arrows).

A head computed tomography scan showed a subarachnoid hemorrhage around the midbrain cistern, that extended to the occipital horns of the lateral ventricles (Figures 1a and 1b). Subsequent CT scans revealed a slight hypodensity at the brainstem.

**Figure 2:**
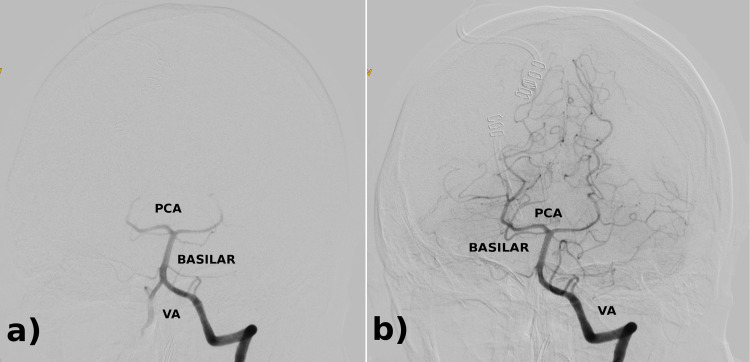
Cerebral angiogram of the left vertebral artery. Cerebral angiogram shows mildly hypoplastic right vertebral artery. Otherwise, patent posterior cerebral circulation. VA: Vertebral Artery; BASILAR: Basilar Artery; PCA: Posterior Cerebral Artery.

A cerebral angiogram showed no observable aneurysms or filling defects (Figure 2).

**Figure 3:**
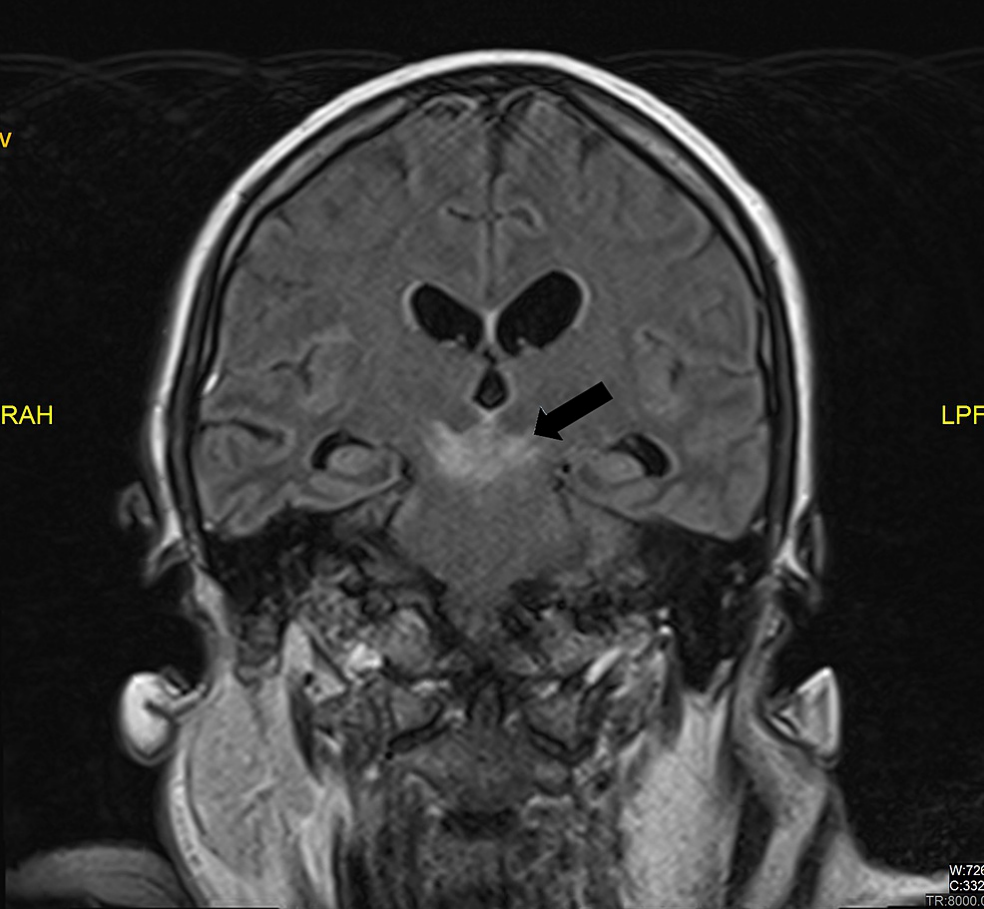
Magnetic resonance imaging of the brain. Brain magnetic resonance T2‐weighted‐FLAIR (fluid‐attenuated inversion recovery) imaging coronal section shows hyperintensity in the midbrain (arrow).

Brain magnetic resonance imaging showed T2- hyperintense lesion at the brainstem likely associated with edema. Consistent with CT findings, there is T2 hyperintensity in the brainstem, being more evident in the midbrain (Figure 3).

**Figure 4:**
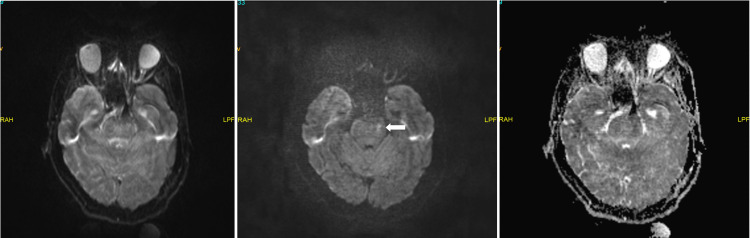
Brain magnetic resonance imaging. Diffusion sequences (b0 and b1000) and apparent diffusion coefficient (ADC) map, respectively. Note the small focus of diffusion restriction on the left cebebral peduncle (arrow).

A punctate diffusion restriction focus is detected in the left cerebral peduncle (Figure 4).

Arterial hypertension was normalized by pharmacological regimen as oral amlodipine 10 mg every 24 hours, oral captopril 25 mg every eight hours, oral doxazosin 4 mg every 12 hours, and oral nimodipine 60 mg every four hours. The patient also received IV fluids 0.9% saline serum 500 ml and sodium chloride 20 ml 21 ml/h. The patient had a good clinical recovery and was able to ambulate with little assistance and consume an oral diet at discharge. His modified Rankin Scale was three.

**Figure 5:**
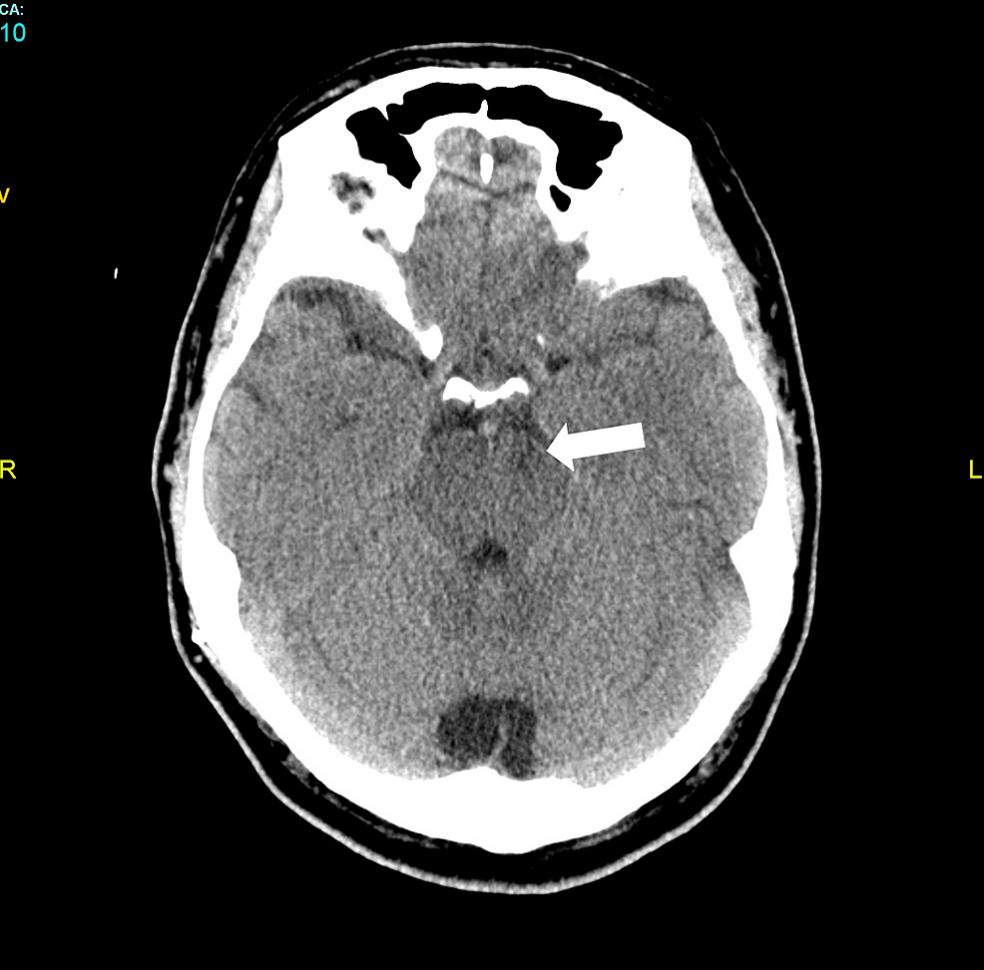
CT without intravenous contrast (axial plane) A small area of encephalomalacia is detected on the left cerebral peduncle (white arrow).

On six months follow up he had residual minimal left third nerve palsy with a modified Rankin scale of one. Follow-up CT head only showed a punctate area of encephalomalacia was recognizable in the left cerebral peduncle, and it corresponded to the area that was restricted in the diffusion sequence of the MRI (Figure 5).

## Discussion

The causes of this syndrome described for the first time in 1996 [7] are controversial and have not yet been fully proven. The most accepted theory is the appearance of a neurotoxic state as a consequence of the inability of the posterior circulation to regulate itself in response to acute changes in blood pressure [8]. There are publications based on perfusion studies in magnetic resonance imaging that observe a greater role in the mechanisms of autoregulatory vasoconstriction, although their conclusions have not been fully validated in subsequent studies [9, 10].

Based on the Fugate and Rabinstein algorithm [11], the mere presence of at least one acute neurological symptom (seizures, encephalopathy/confusion, headache, or visual disturbances) and at least one risk factor (poorly controlled or severe hypertension, kidney failure, immunosuppressive treatment, chemotherapy, eclampsia, or autoimmune disease), is sufficient to suspect a PRES. According to other studies, the symptoms most commonly associated with PRES are seizures and encephalopathy (ranging from drowsiness to coma), with the presence of visual disturbances also being a good predictor of the disease [12,13]. In case of diagnostic doubt, an MRI can be performed, being a suggestive finding for diagnosing the presence of vasogenic edema in a location of the posterior vascular territory. However, there are authors who defend that even the absence of pathological alterations in magnetic resonance imaging does not exclude this diagnosis [14]. The total or partial reversibility of the picture, both at a clinical and radiological level, will also allow the diagnosis to be conclusively established.

The differential diagnosis of PRES is quite broad, being necessary to know the different alternative diagnoses since they have a different prognosis and management. Like PRES, there are other diseases that can present symptoms of headache, decreased level of consciousness, and focal neurological signs. A good example would be subdural, intraparenchymal, or subarachnoid hemorrhages. In this case, it will be of vital importance to get an early diagnosis of its etiology, obtained through a good clinical interview, when possible. It is not only a question of knowing if there has been a recent head injury but also of finding out personal history closely related to these events, such as high blood pressure. Imaging by CT angiography, MR angiography, or angiography will be of vital importance to rule out the existence of aneurysms in the cerebral arteries. Venous sinus thrombosis can also produce symptoms similar to that of PRES. Therefore, when there is suspicion, a venographic vascular study must be performed to rule out this possibility.

Within this differential diagnosis, posterior circulation stroke deserves special attention, since delaying thrombolysis can dramatically affect the patient's prognosis. In the case of infectious encephalitis and meningitis, mainly those related to the herpes simplex virus, an expeditious action can also save lives. This is why some authors propose prophylactic treatment with intravenous acyclovir and antibiotics while the definitive diagnosis is still being sought [14]. Other aetiologies that also affect the white matter, such as chronic hypoxic-ischemic encephalopathy or progressive multifocal leukoencephalopathy, are associated with different clinical contexts, with the distribution of lesions on the MRI being somewhat different.

The case we present constituted a particularly demanding diagnostic challenge due to different circumstances. First, the brainstem location, although previously described, is not the most common as established in previous studies [15,16]. Furthermore, isolated involvement of the brainstem is especially unusual, with only 24 cases having been described in the international scientific literature [17]. Among the intracranial hemorrhages associated with PRES, the subarachnoid is not the most common type [18]. Lastly, although it is assumed that the syndrome is reversible, it may not be totally so, as suggested by our case and studies based on autopsy series [19].

An early diagnosis of PRES is really important to initiate treatment and prevent further complications. For this, it is essential to know what symptoms or radiological findings can be associated with the disease. Although most cases resolve successfully and carry a favorable prognosis, inadequate therapeutic support or delay in treatment may have negative consequences.

## Conclusions

This case highlights the uncommon presentation of midbrain PRES associated with subarachnoid haemorrhage (SAH). It is likely that this pathology is currently underdiagnosed, so the effort to make an accurate diagnosis should be increased, learning to identify the atypical manifestations.
